# Long-Term Assessment of a Set of CO_2_ Concentration Sensors in an In-Use Office Building

**DOI:** 10.3390/s22239403

**Published:** 2022-12-02

**Authors:** Carmen Serrano Lapuente, Héctor Herrada, María José Jiménez, María Nuria Sánchez

**Affiliations:** 1 Energy Efficiency in Buildings R&D Unit, CIEMAT, Avenida Complutense n°40, E-28040 Madrid, Spain; 2 Plataforma Solar de Almería, CIEMAT, Carretera de Senés s/n, E-04200 Almería, Spain

**Keywords:** building energy, performance gap, in situ measurements, CO_2_ concentration, long-term measurements

## Abstract

The measurement of the CO_2_ concentration has a wide range of applications. Traditionally, it has been used to assess air quality, with other applications linked to the experimental assessment of occupancy patterns and air renewal rates. More recently, the worldwide dissemination of COVID-19 establishing a relationship between infection risk and the mean CO_2_ level has abruptly led to the measurement of the CO_2_ concentration in order to limit the spread of this respiratory disease in the indoor environment. Therefore, the extensive application of this measurement outside of traditional air quality assessment requires an in-depth analysis of the suitability of these sensors for such modern applications. This paper discusses the performance of an array of commercial wall-mounted CO_2_ sensors, focusing on their application to obtain occupancy patterns and air renovation rates. This study is supported by several long-term test campaigns conducted in an in-use office building located in south-eastern Spain. The results show a spread of 19–101 ppm, with a drift of 28 ppm over 5 years, an offset of 2–301 ppm and fluctuations up to 80 ppm in instantaneous measurements not related to concentration changes. It is proposed that values averaged over 30 min, using a suitable reference value, be used to avoid erroneous results when calibration is not feasible.

## 1. Introduction

Carbon dioxide sensors are widely used in environmental monitoring, indoor air quality or occupancy estimation. There are a wide variety of carbon dioxide sensor types, such as optical, electrochemical, thermal conductivity and resistance sensors. One of the most used, carbon dioxide sensors based on infrared technology, need to achieve high accuracy at a reasonable cost. The concentration measured by the sensor may differ from the real concentration due to factors such as light source stability, sensor manufacturing technology and ambient temperature and pressure. Therefore, the accurate calibration of carbon dioxide sensors represents a challenge that can provide a basic guarantee for accurate carbon dioxide measurement.

Some studies have focused on the indoor environment, and combined temperature, relative humidity and CO_2_ concentration sensors that can be used in building management systems ensuring adequate ventilation rates. One of the main objectives of health authorities in recent times has been to minimise the risk of airborne transmission of SARS-CoV-2. Villanueba et al. [1] monitored the IEQ conditions in classrooms, assessing one of the control practices implemented through the contingency plans for schools, which is the increasing ventilation rates of the classrooms. To do this, the authors positioned CO_2_ sensors systematically separated from both the students and the walls and at the same height, coinciding with the students’ breathing height. However, it is important to emphasise the relevance of adopting adequate COVID-19-based ventilation strategies to guarantee thermal and acoustic conditions, while keeping CO_2_ concentration levels below the recommended limits [2,3].

In previous studies, Pei et al. [4] investigated the impact of CO_2_ sensors located indoors on the performance of controlled ventilation systems. The authors determined multiple factors that influenced the sensing performance, such as occupancy and ventilation rates and strategy. The optimal spatial distribution of sensors for proper data quality measurements can be performed by applying computational fluid dynamic analysis [5]. Mylonas et al. [6] assessed the limitations and capabilities of indoor environmental sensors by comparing six different wireless sensors tested in a climate chamber. They used high-precision reference instruments to compute any deviation from real conditions. They concluded, as did Petersen et al. [7], that there is no dominant factor related to CO_2_ accuracy dependency. Other authors evaluated the suitability of low-cost Arduino CO_2_ sensors for indoor built environments [8]. CO_2_ concentrations ranging between 400 and 2500 ppm were evaluated and showed non-negligible high deviations in measured values compared to reference ones.

CO_2_-based detection systems have been used in office and residential buildings for occupancy estimation [9]. One of the main advantages of CO_2_-based detection systems over other sensing systems is their ease of implementation in existing building infrastructures [10]. Using CO_2_-based sensing systems, several applications in buildings have been reviewed in the literature, including demand control ventilation [11] and in-line airflow control [12]. A reliable and robust estimation of building occupancy can be performed using the potential of the heterogeneous multisensor fusion approach: CO_2_, temperature, humidity, light, etc. (Kampezidou et al. [13]). The authors proposed a pattern recognition machine methodology in order to identify the room’s binary occupancy state based on limited sensors: CO_2_ and temperature. Esposito et al. [14] tested dynamic neural networks, based on sensor outputs limited to several weeks, for stochastic field calibration of low-cost indicative air quality detection systems, focusing in this case on the estimation of the NO_2_ concentration. Tekler et al. [15] applied a two-step feature selection algorithm identifying the number of Wi-Fi-connected devices and the indoor CO_2_ concentrations as crucial features for predicting occupancy in an office, library and lecture room. The authors also identified the best model performance for each space type implementing a different deep learning architecture in this study.

## 2. Related Work

It is well known that CO_2_ concentration sensors at fine spatio-temporal resolutions typically have an offset that drifts throughout time. Borodinecs et al. [16] reviewed the possible uncertainties in indoor CO_2_ measurements. They concluded that sensors widely available in the market are calibrated mainly by using the general assumption on the outdoor air CO_2_ concentration. They stated that these limitations are critical for practical application in rooms without mechanical ventilation and/or with a variable room occupancy profile.

In addition, usually, the measurements carried out with these sensors show fluctuations that do not correspond to actual CO_2_ concentration variations. So, it is important that manufactures limit the performance time for low-cost sensors based on a standard durability test and that advanced methods of rapid and cost-effective calibration of such sensors be developed.

Spinelle et al. [17] developed a protocol for the evaluation and calibration of low-cost commercial sensors for the monitoring of air pollution. The authors calibrated the sensors tested in the same conditions against reference measurements by regression methods and learning techniques relying on multi-months monitored data. Taking the measurement uncertainty estimated by orthogonal regressions as an indicator, the latter methodology shows better agreement between the values recorded by the sensors and the reference measurements [18]. Mao et al. [19] proposed a deep-learning-based algorithm for rapid calibration of carbon dioxide sensors characterised by high efficiency, accuracy and low cost. As a main result, a back-propagation neural network was chosen as the model. Vajs et al. [20] used machine learning to correct the impact of temperature and relative humidity on low-cost sensors’ accuracy. Other authors analysed the spatial and temporal variability of correction factors obtained from field calibration [21]. They applied three different methods for calibration: daily updated correction factors, corrections based on uniform low concentrations and a Bayesian regression model. As mentioned previously, the behaviour of these sensors can lead to inaccuracies in the assessments based on their measurements. This could be the case in comparisons of raw measurements with certain absolute fixed levels, for example, when these sensors are used to detect whether the CO_2_ concentration in a space has exceeded a certain level. The calculations that contain a subtraction of the measurements carried out by different sensors could also be problematic, for example, in calculations of differences between the CO_2_ concentrations of different rooms or in differences between a room and the exterior. In principle, the calibration of these sensors could be seen as the evident solution to the observed problems, but its cost could be unaffordable in some applications.

Some previous works have applied strategies to overcome the observed problems. One of these successful applications has been the assessment of the occupancy patterns in occupied spaces in office buildings [22,23] and schools [24], from the evolution of the CO_2_ concentration related to the metabolic activity of the users. In these cases, the offset of the sensors did not affect the accuracy of the results, because the analysis was based on differences of measurements carried out by the same sensor and consequently cancelling the offset by means of the difference. The fluctuations were smoothed doing 1-h moving averages. An analogous strategy was applied to assess the infiltration rate of office buildings from raw measurements of the decay of the metabolic CO_2_ in the office buildings just after the users left the rooms [25]. However, in this case, the results were significantly worse than the results obtained using traditional N_2_O as a tracer gas technique, suggesting further research to improve the assessments based on the measurement of metabolic CO_2_.

The novelty of the work reported in this paper is the validation, at an experimental level using long-term campaigns, of the feasibility of using CO_2_ concentration sensors in certain CO_2_-based applications without the need to carry out expensive periodic standard calibrations to mitigate the effects of offset, drifts and parasitic fluctuations in the measurements. This work analyses the mentioned problems of the CO_2_ concentration sensors (offset, drift and fluctuations not caused by changes in the CO_2_ concentration). The behaviour of a set of nine sensors, continuously measuring for a long-term test campaign in an in-use office building, was analysed. From a qualitative point of view, this work confirms that the considered set of CO_2_ concentration sensors behave as expected according to the technical specifications of the manufacturers and previous research works. Additionally, the observed behaviour was quantified for more than 14 years of measurements and two additional benchmark test campaigns. The results highlight the need to calibrate these sensors periodically. Some alternatives based on the conducted work and its findings are suggested to skip the problems related to the lack of calibrations, which are useful for certain applications where standardised calibrations are not feasible.

This document is organised as follows: Section 2 describes the building where the tests were conducted, the experimental set-up, the data analysed and the applied methodology; Section 3 presents and discusses the results; and, finally, Section 4 summarises the conclusions of this work.

## 3. Materials and Methods

The materials used in this work are constituted by the building described in Section 3.1, the measurement devices are described in Section 3.2 and the data are described in Section 3.3. The methods are described in Section 3.4.

### 3.1. Monitored Office Building

The building where the tests were conducted corresponds to an office building located at the Plataforma Solar de Almería (PSA), in the Tabernas desert, in Almería, Spain. This area is characterised by a cold desert climate, with low annual rainfall, mean annual temperatures around 18 °C and high daily thermal oscillations [26]. Its climatic zone, according to the Köppen–Geiger classification, is BWk.

**Figure 1:**
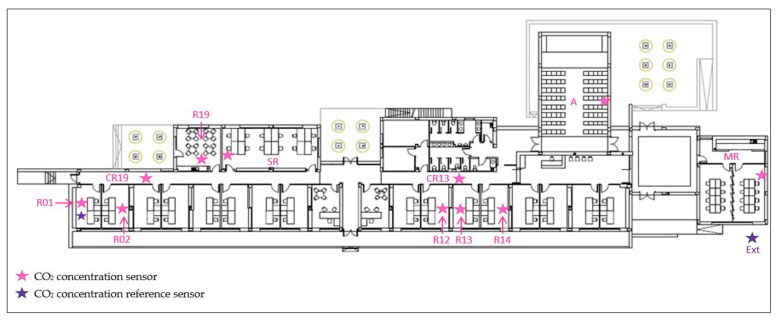
Overview of the building plant indicating the spaces where the CO_2_ sensors are installed. Sensor nomenclature is showed.

The building is distributed along a longitudinal axis on a single floor with an area of about 1000 m^2^. The predominant facades are oriented to the North and South. The interior layout of the building is divided into different volumes separated by a corridor. The offices are located mostly on the south side. The building is continuously monitored under real conditions of use. The design and implementation of monitoring systems both inside and outside the building allow a global energy assessment of the building to be carried out [27]. Figure 1 shows an overview of the building plant. More information is included in Olmedo et al. [28].

### 3.2. Measurement Devices

Three types of sensors were used in the considered building. These types of sensors are coded as types 0, 1 and 2 in this work, as indicated in Table 1.

The type 1 and 2 sensors are similar regarding cost, accuracy and other metrology characteristics. These type 1 and 2 sensors were used to measure the indoor CO_2_ concentration. The type 0 sensors have better accuracy and are more expensive than the type 1 and 2 sensors. These type 0 sensors were used to measure the outdoor CO_2_ concentration. Additionally, one type 0 sensor was installed as a reference in an office of the monitored building, and it was used as a reference considering that its accuracy is remarkably better than the accuracy of the other types of sensors.

#### 3.2.1. Sensors Built into the Monitoring System of the Building Being Continuously Monitored

The office building has a comprehensive monitoring system that has been running continuously since 2008.

A selection of representative offices were identified and monitored in detail. This monitoring includes measurement of the driving variables that are necessary to obtain all the energy contributions to the office. This comprises measurements in the selected offices, their adjacent spaces and outdoors. The work reported in this paper is focused on the CO_2_ concentration measured in these indoor and outdoor spaces.

Initially, the CO_2_ concentration in the offices of the building was being measured using type 1 sensors since 2008. When any of these sensors was damaged or malfunctioned, it was replaced by a sensor of the same type. Since 2016, some of these replacements introduced type 2 sensors.

A type 0 sensor was being used to measure the outdoor CO_2_ concentration. It was installed outdoors, on a meteorological mast installed on the roof of the building. Additionally, one type 0 sensor was installed as a reference in an office, Room 1, of the monitored building, and it was used as a reference considering that its accuracy is remarkably better than the accuracy of the other types of sensors.

All the type 1 and 2 sensors installed in the building were replaced in June 2022 by a set of type 2 sensors calibrated through a benchmark test conducted in 2021. The sensor nomenclature, position and type are indicated in Figure 1.

#### 3.2.2. Additional Test Set-Up and Measurement Campaigns

**Figure 2:**
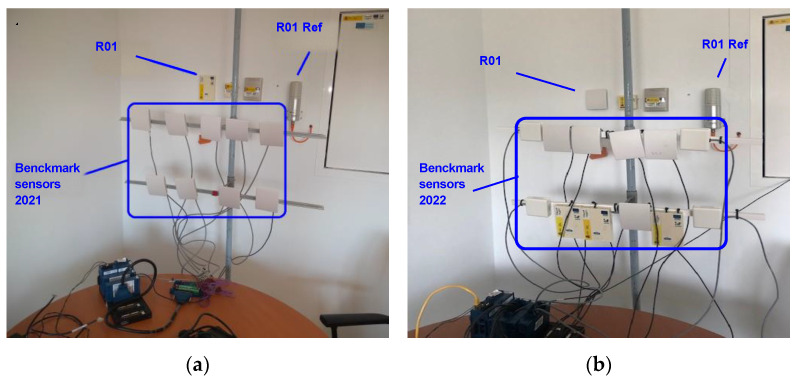
(**a**) Benchmark set-up 2021. (**b**) Benchmark set-up 2022.

Two additional test campaigns were specifically conducted from August 2021 to October 2021 and from August 2022 to September 2022, complementing the monitoring data recorded by the built-in data acquisition system of the building. These tests campaigns were carried out in the office named as Room 1 (R01). There, a set of sensors of the CO_2_ concentration were installed in the same benchmark test, as shown in Figure 2. This arrangement was close to the CO_2_ type 0 and type 1 sensors, already installed in the built-in monitoring system of the building, whose measurements were being continuously recorded by this monitoring system. This set-up aims to ensure that all the sensors in the benchmark test as well as the two sensors already installed in the room are exposed to the same CO_2_ concentration.

The benchmark test conducted from August 2021 to October 2021 included nine type 2 sensors. All the sensors calibrated through the benchmark test conducted in 2021 were installed in the built-in system of the building on 26 July 2022. These sensors replaced six type 1 sensors and one type 2 sensor that were installed until then. The two remaining sensors were installed in the monitoring built-in system, measuring the CO_2_ concentration in the auditorium and in the meeting room, respectively, where this variable had not been previously measured.

The benchmark test conducted between August and September 2022 was analogous to the previous one but included four CO_2_ sensors that were removed from the other rooms in the building on 26 July 2022 and four CO_2_ type 2 sensors never used before.

### 3.3. Data

Section 3.3.1 and Section 3.3.2 hereafter describe the data used to carry out the work reported in this paper that were obtained respectively from the two different data sources described in Section 3.2: built-in monitoring system (Section 3.2.1) and benchmark test (Section 3.2.2).

#### 3.3.1. Continuous Monitoring

Continuous monitoring has been running since 1 August 2008. Measurements have been read and recorded every minute. Data corresponding to more than 14 years are available and have been used in this work. The availability of such a long test period provides robustness to the applied analysis approach.

All sensors have provided continuous records throughout the 14 years; however, there are some periods of time when data are missing due to sensor damage, malfunction, repairs or substitutions. For the reference sensors, on the one hand, the exterior reference sensor (Ext) was installed in 2010 and stopped recording data in 2017. On the other hand, the measurements of the interior reference sensor of Room 1 (R01 Ref) cover a period from 2013 to 2020. Consequently, both sensors only provide data simultaneously from 2013 to July 2017.

Records are saved in text format files from the data acquisition system to later extract them using data processing tools.

#### 3.3.2. Intensive Measurement Campaigns: Benchmark Tests

Two intensive test campaigns were conducted between August and October 2021 and August to September 2022 in Room 1. Different occupancy patterns can be distinguished in these periods:

- First half of August 2021 and 2022, first half of September 2021 and first week of September 2022: Room 1 was empty, but other rooms of the building could be occupied.
- Second half of August 2021 and 2022: There were no occupants in the entire building except for the 2 or 3 last days of August when some offices could be occupied.
- Since 16 September 2021 and since 5 September 2022: Room 1 and all the rooms in the building are regularly in use along the working schedule of the centre (Monday to Friday, 8:30 to 16:30 except 1 h that users take to have lunch).

### 3.4. Data Analysis

A systematic analysis including the following aspects was conducted:

1. Analysis of the agreement between the measurements obtained using the two type 0 sensors and the time evolution of this agreement, discussing the suitability to use them as a reference for calibration.
2. Spread between the measurements recorded by the different sensors and offset of each sensor regarding the one taken as a reference. The following set of sensors was analysed:
  - Built-in sensors for different years in periods when the building is empty
  - Benchmark sensors for the test campaigns conducted in 2021 and 2022
3. Trend shown over time by the spread between the measurements recorded by the different built-in sensors in the building.
4. Adjust of the measurements of the benchmark with respect to the reference sensor considering the minutely recorded data and filtered series gathered applying different average periods: 2 min, 5 min, 10 min, 30 min, 1 h, 2 h and 1 day.
5. Analysis of the dependence of the adjustment quality and the average period.

These analyses of the mentioned aspects are described in detail in Section 3.4.1 and Section 3.4.2 hereafter.

#### 3.4.1. Assessment of the Spread and Offset of the Measurements and Their Drift over a Long-Term Period

Considering the type 0 sensors (Ext and R01 Ref) as potential reference sensors, their difference and the drift of this difference from 2013 to 2017 were quantified by subtracting the mean of the first and the mean of the second. Each one of these values corresponds to the mean of the daily mean values of the CO_2_ concentration on days when no occupancy is assured. Data were selected for summer periods, when the building is closed due to holidays, winter periods concerning Christmas vacation and, for the year 2016, a set of weekends. The number of days used for each period varies from a minimum of 7 days to a maximum of 19.

To quantify all sensors’ spread and its drift throughout the 14-year period, their standard deviation was considered. The values used in the calculation correspond to the mean of the daily mean values from the days in August when the building is not occupied for all available sensors.

A calibration of all the indoor sensors regarding the sensor R01 Ref was conducted for non-occupancy periods during the summer holidays. The only exception is the year 2020, for which a period of the lockdown in Spain due to the pandemic SARS-CoV-2 was selected, compensating for the lack of data in summer. To calibrate the sensors, firstly, the mean of the data registered by each one was calculated. Secondly, the mean of the reference sensor and the mean of each sensor were subtracted, obtaining different offsets. Finally, the calibrated value was the result of adding the offset of each sensor to its originally registered data. The same process was applied to the filtered data at 2 min, 5 min, 10 min and 30 min.

#### 3.4.2. Signal Smoothing and Identification of the Suitable Averaging Step

To smooth the peaks resulting from the CO_2_ concentration signals measured every minute, the means for intervals of 2 min, 5 min, 10 min, 30 min, 1 h, 2 h and 1 day were calculated.

The benchmark sensors placed in Room 1 were calibrated regarding the R01 Ref sensor through a linear regression. Periods containing occupancy and non-occupancy, from 28 September to 13 October 2021, were used. The chosen parameter under consideration was the linear regression coefficient (r^2^), calculated for raw data and for the previously mentioned average intervals.

A plot representing r^2^ versus the mean interval was created. If an asymptotic trend was observed, i.e., an increase in the coefficient was not significant compared to the increase in timing, the appropriate average step was easily identified.

## 4. Results

The obtained results are described in Section 4.1 and Section 4.2 hereafter.

### 4.1. Assessment of the Offset in the Measurement and Its Drift over a Long-Term Period

**Figure 3:**
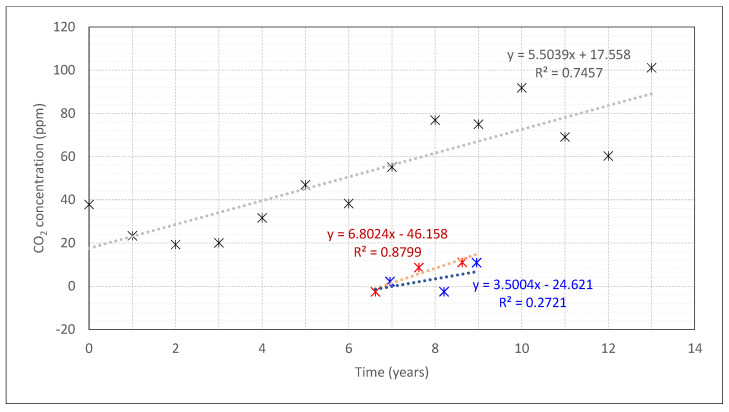
Difference in daily mean values of the CO_2_ concentration between Ext and R01 Ref sensors during unoccupied periods of summer (red) and winter (blue), and spread of the measurements (grey) obtained as the standard deviation of the mean daily mean values of the CO_2_ concentration between R13, R01, R02, R12, R13, R14, SR, CR13, CR19 and R01 Ref CO_2_ sensors during unoccupied periods in August. The 0 in the *x*-axis corresponds to the year 2008.

The difference between the reference sensors and the drift (Ext and R01 Ref) from 2013 to 2017 are summarised in Table 2 and Figure 3. It must be considered that the origin of the *x*-axis of this figure corresponds to the year 2008 and the red and blue points represent calculations based on summer and winter periods, respectively. The observed differences were low (Table 2), starting around 2 ppm for 2014 and showing an increasing tendency with time (Figure 3). However, the values remained low even for the last years when comparison was possible (2016–2017). These observations led us to assume a low difference also before 2014. Considering this issue, the exterior device was used as a reference when the indoor reference sensor was not available, which was also before 2014.

**Figure 4:**
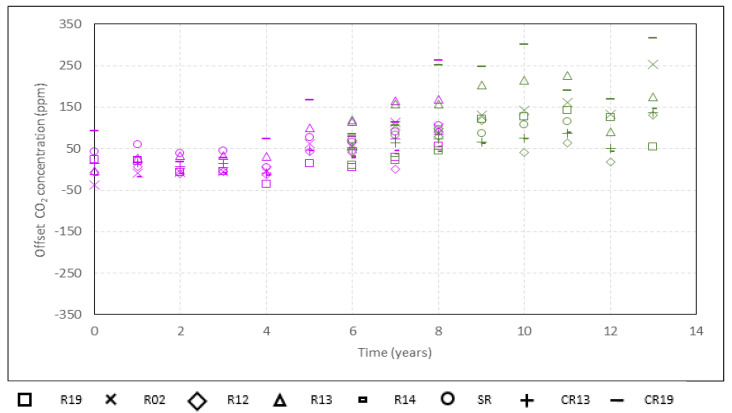
Offset regarding the reference sensor for all the sensors, considering periods when the building was not being used from 2008 to 2021: periods of each year in August and March 2020. From 2008 to 2016, the reference sensor is Ext (purple). Since 2014, the reference sensor is R01 Ref (green). The 0 in the *x*-axis corresponds to the year 2008.

The spread and the drift of the spread of all the sensors are shown in Figure 3. The drift of the offset of all the sensors regarding the exterior until 2016 and regarding R01 Ref since 2014 is shown in Figure 4. The spread, offsets and their drift are summarised in Table 3 and clearly increased. The spread went from 19 to 101 ppm, and an average drift in the spread of 28 ppm per 5 years was identified. The offset ranged from 2 to 301 ppm.

### 4.2. Signal Smoothing and Identification of the Suitable Averaging Step

**Figure 5:**
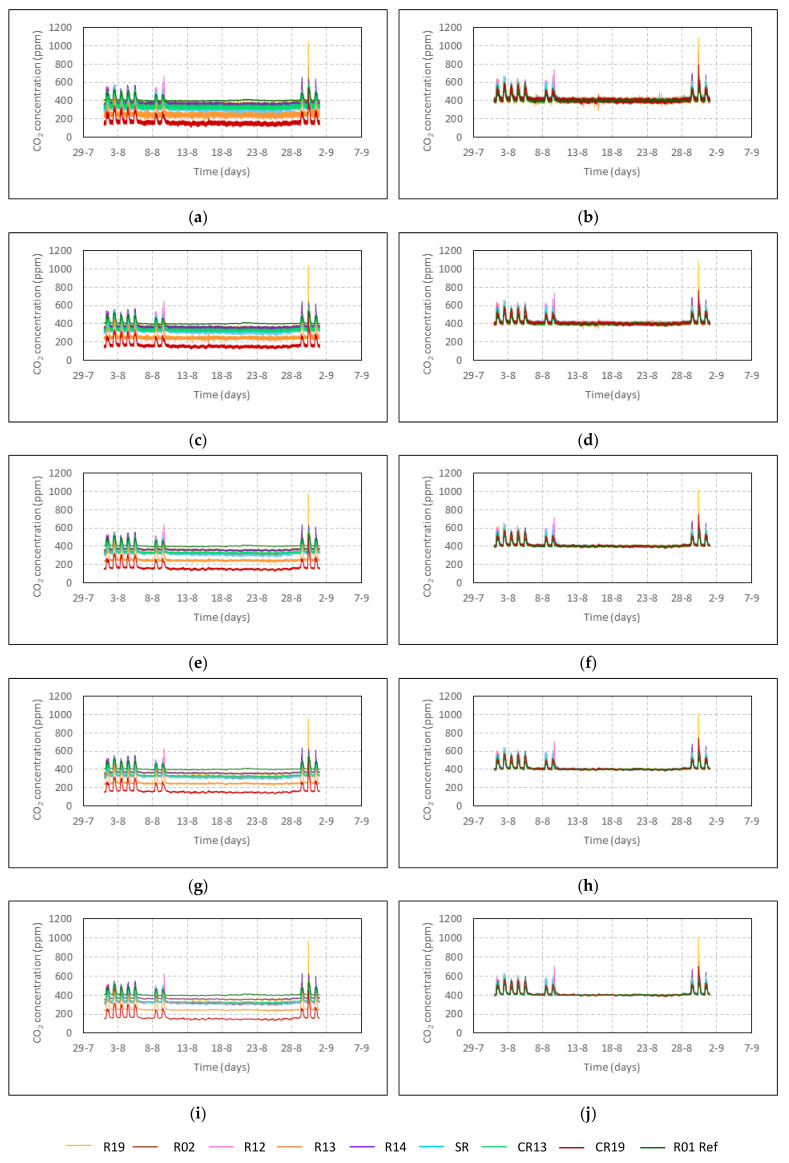
Summer 2016 as an example. The 14 years show similar behaviour. Raw data (**a**) and data averaged at intervals of (**c**) 2 min, (**e**) 5 min, (**g**) 10 min and (**i**) 30 min and calibrated measures with sensor R01 Ref for raw data (**b**) and the same average intervals (**d**,**f**,**h**,**j**), respectively.

Once the measurements were corrected from the offset, oscillations up to 80 ppm were shown by the raw data minutely recorded (Figure 5b). When all the sensors were filtered applying different average intervals, the signal improved as its noise remarkably reduced. This behaviour can be observed in Figure 5. This figure also shows an improving accordance between all the indoor measurements and the reference sensor as the average interval increased. The oscillations in the measurements reduced as the average interval increased, being 20 ppm for 30 min averages (Figure 5j).

**Figure 6:**
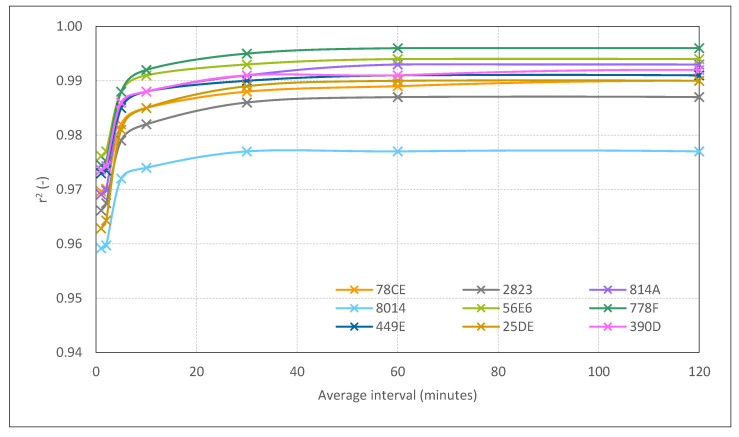
Benchmark set-up 2021. The r^2^ value of the lineal regression of the measurements (serial numbers: 78CE, 2823, 814A, 8014, 56E6, 778F, 449E, 25DE, 390D) with sensor R01 Ref. Values obtained for raw data minutely recorded and average intervals of 1 min, 2 min, 5 min, 10 min, 30 min, 1 h and 2 h.

Considering the 2021 benchmark, an enhancement of r^2^ was observed with higher values of average intervals (Table 4). Figure 6 shows that the curve started to flatten, reaching its maximum value approximately at the 30 min interval. In addition, a decrease in r^2^ was registered when using 1-day means. The results of the linear regression parameters considering 30 min averages are summarised in Table 5. The spread of the measurements and their offset before and after being adjusted using the parameters from these regressions are shown in Table 6.

According to the observed behaviour, 30 min was selected as a suitable period in which the increase in r^2^ did not imply a significant improvement with respect to longer time intervals (Figure 6).

## 5. Discussion

According to the results reported in Section 4, the following issues are characteristic of the performance of the considered CO_2_ sensors:

- A certain offset that is different among distinct sensors of the same type.
- The measurements obtained using different sensors shows a certain spread.
- The individual offset and the spread between the sensors drift over time.
- The instantaneous measurements showed large fluctuations that are not related to these changes in the CO_2_ concentration.

This behaviour can lead to uncertain results in a calculation containing a subtraction incorporating measurements recorded with different sensors in the following cases:

- Comparisons of the raw measurements with certain absolute fixed levels, for example, if these sensors are used to detect whether the CO_2_ concentration in a room has exceeded a certain level
- Calculations that contain a subtraction of measurements from different sensors, for example, in calculations of differences between the CO_2_ concentrations in different rooms or differences between a room and the outside

The results highlight the need to periodically calibrate these sensors, with some exceptions for certain applications where standardised calibrations are not feasible or their costs are not affordable. The following strategies based on the conducted work and its findings are suggested to avoid wrong results due to the identified problems:

- The use of values averaged over a certain period instead of instantaneous measurements. This work reveals that 30 min averaging intervals are enough to significantly reduce unexplained sensor fluctuations. This strategy is effective combined with any of the two following options.
- Detection of variations in CO_2_ measurements carried out by one sensor regarding a reference value obtained by itself. This technique has been successfully applied in several previous published works [22,23,24,25].
- Calibrations of the sensors at least regarding a reference sensor just before each test campaign.

These alternatives extend the usefulness of the type of sensors analysed to a wide range of applications, avoiding the excessive cost of test campaigns.

## 6. Conclusions

The behaviour of a set of nine sensors, continuously measuring for a long-term test campaign in an in-use office building, was analysed. From a qualitative point of view, this work confirms that the considered set of CO_2_ concentration sensors behaves as expected according to the technical specifications of the manufacturers and previous research works. Additionally, the observed behaviour was quantified for more than 14 years.

When periodic calibrations are not conducted, the measurements are affected by a certain offset, where the electric measurement is transformed to the CO_2_ concentration using the default transformation function given by the manufacturer. This offset is different among different sensors of the same type. This work found an offset from 2 to 301 ppm. A spread from 19 to 101 ppm was identified. The individual offset and the differences between the sensors drift over time. A drift of 28 ppm was observed over 5 years in the spread of the different sensors. The assessed performance can lead to uncertain results in a calculation containing a subtraction of measurements recorded with different sensors. The identified behaviour stresses the need to calibrate the sensors just before each test campaign or at least to identify an offset of each used device regarding a reference sensor if standardised calibrations are not feasible or their costs are unaffordable. Another alternative applied in previous research, valid in certain applications, is to skip these calibrations detecting variations in the CO_2_ measurements by one sensor regarding a reference value obtained by itself [22,23,24,25].

Additionally, instantaneous measurements showed large fluctuations, up to 80 ppm, that are not related to these changes in the CO_2_ concentration. This work revealed that using 30 min averaging intervals significantly reduces unexplained sensor fluctuations in the analysed test campaigns, lowering the fluctuations to 20 ppm approximately. According to this result, assessing the optimum average interval and filtering the raw data by the determined optimum averaging interval are recommended as part of the pre-processing of CO_2_ concentration measurements.

The suggested strategies to overcome the identified problems can be applied to avoid periodic standardised expensive calibrations, extending the usefulness of the type of sensors analysed to a wide range of applications.

Some of the potential applications of the findings of this work are the experimental assessments of occupancy patterns and air renovation rates from the measurement of the metabolic CO_2_ in the built environment. In this context, some previous research works have avoided the calibration of these sensors by detecting variations in CO_2_ measurements carried out by one sensor regarding a reference value obtained by itself [22,23,24,25]. Further research on these applications incorporating the findings reported in this paper can contribute to improving the results of these previous works.

## Figures and Tables

**Table 1:** Types of sensors used in this analysis and summary of their characteristics.

Sensor Features	Type 0: GMP343 VAISALA Vantaa, Finland	Type 1: GMW115 VAISALA Vantaa, Finland	Type 2: EE800-M11A6VN0 E+E ELEKTRONIK Engerwitzdorf, Austria
Measurement range	0–2000 ppm	0–2000 ppm	0–2000 ppm
Accuracy after factory calibration with 0.5% accurate gases	±2.5% of reading		
Accuracy (including repeatability, non-linearity and calibration uncertainty)		±(50 ppm CO_2_ + 3% of reading)	
Accuracy at 25 °C and 1013 mbar			<±(50 ppm + 2% of measuring value)
Long-term stability	<±2% of reading/year	±100 ppm CO_2_/5 years	
Response time (T_90_)	30 s	1 min	typ. 110 s
Temperature dependence, typical	±0.3% (<500 ppm CO_2_) and ±1% (>500 ppm CO_2_)	−0.35% of reading/°C	±(1 + CO_2_ concentration (ppm)/1000) ppm/°C (−20 to 45 °C)
Pressure dependence, typical	700–1300 hPa: ±2% (<500 ppm CO_2_) and ±3% (>500 ppm CO_2_)	+0.15% of reading/hPa	
Warm-up time	full accuracy ± 0.5%: 10 min; full accuracy: 30 min	1 min, 10 min for full specification	
Calibration interval			>5 years
Product lifetime		>10 years	

**Table 2:** Difference between the outdoor and the reference CO_2_ concentration measurements, considering their averages in periods when both records were available and the building was not occupied.

Period	CO_2 ext_ − CO_2 ref_ (ppm)
14 August 2014–16 August 2014 13 September 2014–14 September 2014 20 September 2014–21 September 2014	2.2
24 December 2014–31 December 2014	−2.6
13 August 2015–30 August 2015	8.6
6 February 2016–7 February 2016 13 February 2016–14 February 2016 28 February 2016 5 March 2016–6 March 2016 12 March 2016–13 March 2016 19 March 2016–20 March 2016	−2.5
10 August 2016–28 August 2016	11.1
24 December 2016–1 January 2017	10.9

**Table 3:** Offset regarding the reference sensors and spread of all the measurements, considering periods of each year in August and March when the building was not being used from 2008 to 2021. From 2008 to 2016, the reference sensor is Ext (second row when both references are available). Since 2014, the reference sensor is R01 Ref (first row when both references are available).

	2008	2009	2010	2011	2012	2013	2014	2015	2016	2017	2018	2019	2020 *	2021
Spread (ppm)	38	23	19	20	32	47	38	55	77	75	92	69	60	101
Offset R02 (ppm)							61	105	89	131	142	161	134	253
	−37	−8	−7	−4	−2	62	57	114	100					
Offset SR (ppm)							71	83	96	86	109	115	-	-
	43	60	39	45	5	77	66	91	107					
Offset R12 (ppm)							45	-	79	117	41	63	18	131
	−7	6	−11	5	−13	43	40		90					
Offset R13 (ppm)							120	157	157	203	215	226	91	175
	−4	28	34	34	32	100	116	165	168					
Offset CR13 (ppm)							53	65	74	65	76	87	51	136
	13	16	6	13	−9	46	49	73	85					
Offset R14 (ppm)							32	37	43	62	73	88	42	146
	−14	−18	−11	−9	−15	46	28	46	54					
Offset R19 (ppm)							10	21	45	122	127	143	125	54
	23	20	−8	−5	−36	15	5	30	56					
Offset CR19 (ppm)							84	106	252	248	301	191	168	317
	93	29	19	28	73	198	80	114	263					

* Data from 2020 correspond to March due to the lack of data in summer.

**Table 4:** Relationship between the average period and the agreement between the reference and other sensors. Assessment based on r^2^. Benchmark test conducted in August 2021.

Sensor	1 min	2 min	5 min	10 min	30 min	1 h	2 h	1 day
78CE	0.970	0.970	0.982	0.985	0.988	0.989	0.990	0.985
2823	0.966	0.967	0.979	0.982	0.986	0.987	0.987	0.979
814A	0.969	0.970	0.985	0.988	0.991	0.993	0.993	0.993
8014	0.959	0.960	0.972	0.974	0.977	0.977	0.977	0.959
56E6	0.976	0.977	0.988	0.991	0.993	0.994	0.994	0.991
778F	0.974	0.975	0.988	0.992	0.995	0.996	0.996	0.995
449E	0.973	0.974	0.985	0.988	0.990	0.991	0.991	0.983
25DE	0.963	0.964	0.981	0.985	0.989	0.990	0.990	0.982
390D	0.974	0.974	0.986	0.988	0.991	0.991	0.992	0.983

**Table 5:** Correction function obtained from the benchmark test conducted in 2021.

Sensor Serial Number	*a*	*b* (ppm)	*r* ^2^
78CE	0.95	175	0.970
2823	0.97	140	0.966
814A	1.00	177	0.969
8014	0.98	202	0.959
56E6	0.99	143	0.976
778F	1.00	173	0.974
449E	1.00	108	0.973
25DE	0.97	112	0.963
390D	0.98	91	0.974

**Table 6:** Offset regarding the reference sensor and spread of all the measurements, considering the benchmark test conducted in 2021.

	Raw Data	Corrected Data
	(ppm)	(ppm)
Spread	57.3	1.05
SN 2823	130	1.70
SN 56E6	139	0.73
SN 449E	108	0.07
SN 25DE	100	0.54
SN 778F	173	0.10
SN 78CE	156	1.24
SN 8014	197	1.29
SN 390D	85	2.16
